# Shared molecular pathways in pediatric genetic epilepsies: Insights from a single‐center cohort of 80 patients

**DOI:** 10.1002/epi4.70357

**Published:** 2026-10-03

**Authors:** Laura Hecher, Mathias Woidy, Franziska Plein, Tatjana Bierhals, Leonie von Elsner, Theresia Herget, Maja Hempel, Fanny Kortüm, Lev Grinstein, Julia V. Cramer, Jessika Johannsen, Jonas Denecke

**Affiliations:** ^1^ Department of Pediatrics University Medical Center Hamburg‐Eppendorf Hamburg Germany; ^2^ German Center for Child and Adolescent Health (DZKJ), Partner Site Hamburg Hamburg Germany; ^3^ Institute of Human Genetics University Medical Center Hamburg‐Eppendorf Hamburg Germany; ^4^ Present address: Institute of Human Genetics Heidelberg University Heidelberg Germany

**Keywords:** gene ontology, genetic epilepsy, molecular pathways, pediatric epilepsy, whole‐exome sequencing

## Abstract

**Objective:**

Genetic epilepsies in childhood are highly heterogeneous, and approaches to identify shared biological mechanisms across distinct genetic etiologies remain limited. We aimed to investigate whether genes associated with pediatric genetic epilepsies in our heterogeneous cohort converge on common functional pathways.

**Methods:**

We conducted a single‐center retrospective study involving 80 children who met the International League Against Epilepsy (ILAE) criteria for epilepsy with a confirmed monogenetic etiology, with or without other neurological conditions. These children were part of a cohort of pediatric patients with a broad spectrum of neurological disorders that had been genetically diagnosed using trio‐based whole‐exome sequencing. Clinical data were systematically collected. Gene Ontology (GO)‐based enrichment analysis was performed to explore functional convergence.

**Results:**

The cohort comprised 80 patients (39% female, 61% male) with a median age at seizure onset of seven months and included 66 distinct disease‐causing genes and four pathogenic copy number variants. Despite marked genetic heterogeneity, enrichment analyses revealed convergence in functional pathways related to synaptic signaling, membrane channels and transport processes, and neurodevelopmental and neuronal plasticity pathways. Age‐stratified analyses suggested variation in the relative contribution of these pathways across developmental stages, while no significant differences were observed with respect to neurodevelopmental delay or neuroimaging abnormalities.

**Significance:**

These findings demonstrate shared molecular pathways across clinically and genetically diverse pediatric neurological conditions associated with epilepsies and support a pathway‐oriented perspective that may complement gene‐based classification and inform future therapeutic strategies.

**Plain Language Summary:**

Childhood epilepsies caused by genetic changes can arise from many different genes, making them difficult to understand and classify. In this study, we analyzed 80 children with genetically confirmed epilepsy and found that, despite different genetic causes, many of the affected genes are involved in similar biological processes, particularly those related to brain signaling and development. These findings suggest that different genetic epilepsies may share common underlying mechanisms. A better understanding of these shared pathways may help improve how epilepsy is classified and treated in the future.

**Key points**

- Shared molecular pathways are identified across 66 genes and 4 CNVs in pediatric epilepsies.
- Convergent pathways include synaptic signaling, ion channel function, and neurodevelopment.
- Early‐onset epilepsies are linked to synaptic and developmental processes, shifting toward neuronal maturation in later‐onset cases.

## 1 INTRODUCTION

Epilepsy is one of the most common neurological disorders, defined by unprovoked seizures, which occur recurrently or have a significant risk of recurrence.^1^ It affects 4–10 per 1000 individuals worldwide, with the highest incidence in infancy and childhood.^2^ Therapy is particularly challenging in childhood due to the dynamic development of the brain.^3^ Epilepsy itself places a significant burden on patients and families due to the need for regular doctor visits and hospitalizations.^4^ Additionally, depending on the underlying disease, epilepsy carries a risk of impaired physical and/or mental abilities and premature death.^5^

In the past, the etiology of epilepsy often remained unclear, and the term “idiopathic” was frequently part of the diagnosis. In recent years, the idea that the manifestation of epilepsy without an exogenous cause requires a genetic predisposition has gained acceptance. Consequently, a recent report published by the ILAE (International League Against Epilepsy) Commission on Classification and Terminology defines six categories of etiologic categories of epilepsy: structural, genetic, infectious, metabolic, immune, and unknown.^6^ Notably, a patient's epilepsy may be classified into more than one etiologic category, as for example, structural or metabolic etiologies may have a genetic basis.^6^ Following two national population studies, the overall genetic etiology in epilepsy can be assumed to be about 30–40%.^7^, ^8^ A recent study of childhood and youth epilepsies classified 53% as presumably genetic and 10% as proven genetic epilepsies.^9^ In about 70–80% of epilepsy cases, genetic variants are considered to be at least partly responsible for the onset of symptoms, caused by complex interactions between genetic and environmental factors.^10^

Since the application of Next‐generation sequencing (NGS) methods such as targeted gene panels, whole‐exome sequencing (WES), and whole‐genome sequencing (WGS), the number of genes associated with epilepsy has significantly increased.^11^, ^12^, ^13^ At least 134 genes causing epilepsy as a monogenetic disorder were reported until 2024.^2^ Initially, predominantly genes coding for ion channels and neurotransmitter receptor subunits were identified, but increasingly genes involved in synaptic function, gene regulation, or brain development are identified as a monogenetic cause of epilepsies,^14^, ^15^ emerging the role of neuronal network function in the genesis of epileptic activity. Nevertheless, the complexity of the molecular mechanisms involved in epilepsy is poorly understood but crucial for its treatment and management. Computational literature reviews of established epilepsy‐associated genes and in silico efforts to identify biological groups and networks underline the need for a better understanding of the underlying molecular mechanisms involved in epilepsy.^12^, ^16^, ^17^

Gene Ontology provides a standardized, hierarchical framework for categorizing gene products based on their specific molecular functions, cellular locations, and involvement in broader biological processes. Enrichment analysis leverages this framework to identify statistical overrepresentation of any functional category within a clinical dataset, effectively translating high‐throughput genomic results into actionable biological insights and therapeutic targets.^18^, ^19^

In this study, we analyzed 80 genetic findings from children with a monogenetic disease and the diagnosis of epilepsy who thus meet the criteria for confirmed genetic epilepsy according to the latest ILAE classification, regardless of the mechanism of epileptogenesis and type of epilepsy. Thus, by including different pathomechanisms of epileptogenesis, such as channelopathies, brain malformations, and genetically caused bleeding events, we aimed to better understand the functional groups and molecular pathways of gene products associated with childhood epilepsy using Gene Ontology.

## 2 METHOD

### 2.1 Patient inclusion criteria

The study was performed as a single‐center study over 9 years.

From 2014 to 2023, 704 children with heterogeneous neurological disorders underwent a comprehensive diagnostic work‐up including whole‐exome sequencing (WES), performed as trio analysis whenever feasible and as duo analysis in selected cases. WES was considered in patients with the inclusion criteria: neurological symptoms, suspected genetic etiology, no specific provisional genetic diagnosis, and signed consent.

Patients with a clinical diagnosis suggestive of a specific genetic disorder that was routinely addressed by targeted diagnostic testing (e.g., single‐gene or gene‐panel testing) were not routinely referred for WES and were therefore not represented in this cohort.

The diagnostic program routinely included electroencephalography (EEG) and cranial magnetic resonance imaging (cMRI, 1.5 or 3‐Tesla). Detailed phenotyping, WES, variant processing, and data interpretation through a multidisciplinary team were performed as reported before.^20^ Out of this cohort, patients with epilepsy were identified by searching our institutional database for “epilepsy” or “seizures,” and fulfillment of the ILAE‐criteria for epilepsy was verified. Children with epilepsy associated with a monogenetic disorder (likely pathogenic (class 4) or pathogenic (class 5) variant according to ACMG criteria) and no other non‐genetic explanation for epilepsy, such as cerebral hemorrhage in premature infants, were included. The final study cohort comprised n = 80 patients. The study was approved by the ethics committee of the Hamburg Medical Association (PV7038). Informed parental consent was mandatory for inclusion.

### 2.2 Genotype categorization

Copy‐number‐variations (CNVs) and genes identified to harbor a pathogenic or likely pathogenic variant were categorized into one of three groups: known disease gene curated by OMIM (Online Mendelian Inheritance in Man; An Online Catalog of Human Genes and Genetic Disorders), novel disease gene due to newly published data at the time of diagnosis, or candidate gene at the time of diagnosis, which by now could be reclassified as a (novel) disease gene.

Variants were categorized by type: missense variant, nonsense variant, frameshift variant, in‐frame variant, splice‐site variant, CNV, and by inheritance/zygosity: autosomal dominant inheritance ([AD], heterozygous) (including de novo variants and mosaic variants), autosomal recessive inheritance ([AR], homozygous or compound heterozygous), and x‐linked inheritance ([XL], hemizygous/male and heterozygous/female).

### 2.3 Phenotype documentation

We performed a retrospective analysis of the medical reports (including EEG and cMRI reports) prepared by the Department of Pediatrics, University Medical Center Hamburg‐Eppendorf. In addition, sex, the presence of neurodevelopmental delay (NDD), age at seizure onset, age at inclusion for whole‐exome sequencing, and the presence of alternative explanatory factors for epilepsy identified from the medical history, apart from the genetic finding, were assessed. Symptoms were indexed according to the Human Phenotype Ontology (HPO). NDD is defined as delayed attainment of age‐appropriate developmental milestones in one or more neurodevelopmental domains (including motor, language, cognitive, or social/adaptive functioning).

### 2.4 Functional classification of identified genes/proteins

Functional classification of the identified genes and their gene product was performed using Gene Ontology (GO) with respect to three sub‐ontologies: Molecular function (MF; molecular‐level activities performed by the gene product), biological process (BP; larger processes accomplished by multiple molecular activities), and cellular component (CC; cellular locations in which a gene product performs a function).^19^ GO terms are linked through a relational organization including high‐level, broadly descriptive terms and low‐level, specific terms.

### 2.5 Enrichment analysis based on GO terms

Enrichment analyses were performed using R (version 4.4.1) and the additional package clusterProfiler (version 4.13.4.18.1).^21^ Specifically, the function “enrichGO” was applied, using all genes expressed in brain cell types as the background set (parameter “universe”). To define the background set, expression data were downloaded from the Human Protein Atlas (file rna_tissue_consensus.tsv, downloaded on 2023‐01‐25 from https://www.proteinatlas.org/about/download). A gene was considered expressed in brain cell types when the specified nTPM >1 in at least one brain‐specific tissue (cerebral cortex, olfactory bulb, hippocampal formation, amygdala, basal ganglia, thalamus, hypothalamus, midbrain, cerebellum, pons, medulla oblongata, spinal cord, white matter), resulting in a total of 15 782 genes expressed in the brain. *p*‐values were calculated using a hypergeometric test (over‐representation analysis) and adjusted using the false discovery rate (FDR) method. Fold enrichment (FE) was calculated as the ratio of the gene ratio to the background ratio. FE values >1 indicate overrepresentation of a given GO term relative to the background, whereas values <1 indicate underrepresentation.

Plotting was performed using the package ggplot2 (version 3.5.1). Results were combined across GO sub‐ontologies, and adjusted p‐values (<0.05) were natural log‐transformed.

## 3 RESULTS

### 3.1 Phenotype of the study cohort

We identified 80 unrelated children, 31 girls (39%) and 49 boys (61%), with epilepsy according to ILAE criteria and a neurological disorder, in whom a disease‐causing variant was identified by trio‐whole‐exome‐sequencing. The median age at which whole‐exome‐sequencing was performed was 48 months (range: 2 months – 22 years). 41 (51%) children showed MRI abnormalities. 75 (93%) patients had a neurodevelopmental delay (Table 1). The median age at which seizures were first observed was seven months (range: 6 days – 15 years). We manually divided the children into three groups based on their age at the time of their first seizure. This classification was chosen based on clinical experience regarding infantile epileptic spasms syndrome (IESS): epilepsies that manifest before an IESS – including a neonatal manifestation (group 1), at the typical age of manifestation of an IESS (group 2), and beyond this age (group 3). Group 1 included 27 children (34%) who had their first seizure between 0 and 3 months of age (median age: 2 months). Group 2 included 21 children (26%) who had their first seizures between 4 and 12 months of age (median age: 7 months). Group 3 included 32 children (40%) who had their first seizures after 12 months of age (median age: 36 months) (Table 1).

**TABLE 1:** Clinical and genetic characteristics of the cohort of 80 patients with epilepsy.

Number of patients	Percentage	
31	39%	Female
49	61%	Male

Number of patients	Clinical features	
41	51%	Abnormalities on brain magnetic resonance imaging
75	94%	Neurodevelopmental delay (ND)

Number of patients	Age at onset	
27	34%	0–3 months, Median 2 months (IQR 1–3 months)
21	26%	4–12 months, Median 7 months (IQR 5–10 months)
32	40%	1–15 years, Median 36 months (IQR 17.5–60 months)

Number of genes	Genes	
69	86%	OMIM – known disease gene at time of diagnosis
6	8%	Novel disease gene at time of diagnosis
4	5%	Candidate gene at time of diagnosis

Number of variants	Variant Type	
59	60%	Missense variant
5	5%	Nonsense variant
15	15%	Frameshift variant
3	3%	In‐Frame variant
2	2%	Splice‐site variant
1	1%	Start‐loss variant
1	1%	Intronic Variant
4	5%	Copy‐number‐variation

Number of patients	Inheritance	
**51**	**64%**	**Autosomal dominant inheritance (AD)**
43	54%	Confirmed de novo
3	4%	De novo mosaic
3	4%	Inherited
2	3%	Duo‐exome
**18**	**23%**	**Autosomal recessive inheritance (AR)**
**11**	**14%**	**x‐linked inheritance (XL)**
8	10%	Confirmed de novo
3	4%	Inherited

*Note*: Bold values represent the distribution of inheritance patterns of pathogenic variants within the cohort and are descriptive in nature.

### 3.2 Genotype of the study cohort

In 69 (86%) of the 80 patients, a variant in a known disease gene curated by OMIM or a known disease‐related CNV was identified. In six cases (8%; *SSR4, SON, NKX6‐2, INPP4A, GRIA4, DHX30*), the gene identified was classified as a novel disease gene due to newly published data at the time of diagnosis. In four cases (5%; 2x *PAK1*,^22^ *SLC12A9*,^23^ *PIGU* ^24^) a gene initially classified as a candidate gene was identified, which has been reclassified as a (novel) disease gene based on accumulating evidence, including our published reports of these variants and patients (Table 1).

Among the 80 included patients, we identified 66 different genes as monogenetic diseases causing four CNVs. Eight genes were identified as disease‐causing genes in two unrelated individuals: *CDKL5, CHD2, MYT1L, PAK1, SCN8A, SLC6A1, STXBP1*, and *TRIO*, whereas *KCNQ2* was identified as the disease‐causing gene in three non‐related individuals.

Five of these genes appeared in two different age groups. *CDKL5* (age of onset 3 months and 5 months), *STXBP1* (age of onset 0 months and 7 months), and *SCN8A* (age of onset 3 months and 12 months) were identified in group 1 as well as group 2. *SLC6A1* was identified in groups 2 and 3 (age of onset 12 months and 13 months). *TRIO* was identified in groups 1 and 3 (age of onset 1 month and 5 years). Among 80 patients, 51 (64%) carried heterozygous variants consistent with autosomal dominant inheritance. 43 (54%) of these variants were confirmed de novo, three were inherited (4%), three occurred as de novo mosaic events (4%), and in two duo‐exome cases, de novo status remained unresolved (3%). In 18 (23%) patients, the variants occurred biallelic, indicating an autosomal recessive inheritance. In 11 patients (14%), the variant identified showed an X‐linked inheritance (XL) (hemizygous/male and heterozygous/female), of which eight (10%) were confirmed de novo (Table 1).

Most of the identified variants were missense variants (59 variants out of a total of 98 detected variants [60%]). We identified 5 nonsense variants (5%), 15 (15%) frameshift variants, 3 (3%) in‐frame variants, 2 (2%) splice‐site variants, 1 (1%) Start‐loss variant, 1 (1%) intronic variant, and 4 (5%) CNVs (Table 1).

### 3.3 Gene ontology enrichment analysis of identified genes

To identify biological functions associated with genes contributing to epileptogenesis in our cohort, GO enrichment analysis was performed. We found multiple enriched GO terms.

Based on cellular compartment (GO‐CC) terms, genes/gene products clustered into three groups: (I) Neuronal signal transduction and synaptic compartments with the strongest enrichment for “postsynaptic specialization” (gene count 10, FE = 29, *p* = 0.0014) and “neuronal cell body” (gene count 9, FE = 33, *p* = 0.005). Additional enriched terms included “dendritic spine,” “extrinsic component of synaptic vesicle membrane,” and “presynaptic active zone.” (II) Ion channel and transporter‐associated compartments, with strongest enrichment for “extrinsic component of membrane” (gene count = 6, FE = 97, *p* = 0.003) and “node of Ranvier” (gene count = 3, FE = 799, *p* = 0.003). Additional enriched terms included “ion channel complex” and “transporter complex.” (III) A smaller, but biologically distinct category was represented by “complex of collagen trimers” (*n* = 2, FE = 607; *p* = 0.045), corresponding to the genes *COL4A1* and *COL4A2* (Figure 1, Table S2).

**FIGURE 1:**
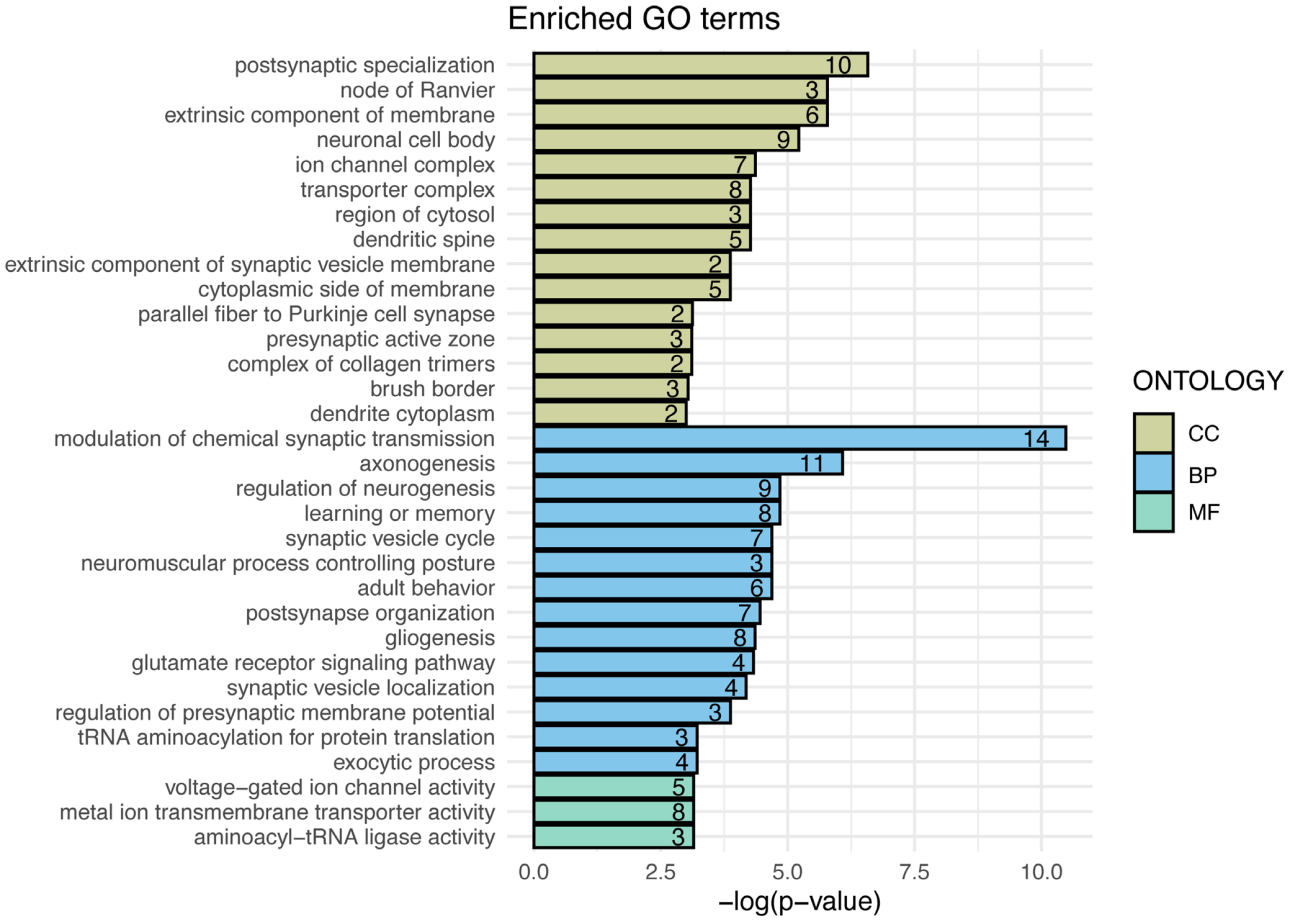
Enriched GO terms of the study cohort. Bar plot showing significantly enriched GO terms among the genes identified in the study cohort. The x‐axis indicates the statistical significance of enrichment (−log *p*‐value), and the y‐axis lists the enriched GO terms. GO terms are grouped by ontology: Cellular component (CC) shown in beige, biological process (BP) shown in blue and molecular function (MF) shown in green. The numbers in each bar depict the genes associated with the respective GO term.

Correspondingly, the most significantly enriched terms in biological process (GO‐BP) were associated with: (I) Synaptic regulation, with the strongest enrichment for “modulation of chemical synaptic transmission” (gene count = 14, FE = 29, p = 2.8 × 10^−5^) and “synaptic vesicle cycle” (gene count = 7, FE = 68, p = 0.009). Additional enriched terms included “postsynapse organization”, “glutamate receptor signaling pathway”, and “regulation of presynaptic membrane potential.” (II) Neurogenesis and neurodevelopment, with strongest enrichment for “axonogenesis” (gene count = 11, FE = 32, p = 0.002), “regulation of neurogenesis” (gene count = 9, FE = 41, p = 0.008), and “gliogenesis” (gene count = 8, FE = 43, p = 0.013). (III) Higher‐order nervous system functions comprised a third group, including “learning or memory” (gene count = 8, FE = 54, p = 0.008) and “adult behavior” (gene count = 6, FE = 98, p = 0.009), and “neuromuscular process controlling posture” (gene count = 3, FE = 801, p = 0.009). At the molecular function (GO‐MF) level, enrichment was observed for “aminoacyl‐tRNA ligase activity” (gene count = 3, FE = 363, p = 0.04), “metal ion transmembrane transporter activity” (gene count = 8, gene ratio = 0.13, p = 0.043) and “voltage‐gated ion channel activity” (gene count = 5, FE = 98, p = 0.043), all directly attributable to membrane channel‐mediated ionic transport and protein synthesis (Figure 1, Table S2).

We next examined whether the enriched terms exhibited a specific age‐of‐onset pattern of seizures. Cellular compartments linked to earlier onset of seizures were primarily those related to membrane channels and transport processes (“node of Ranvier,” median age = 1.5 months; “ion channel complex,” 2.5 months; “transporter complex,” 3 months;), while compartments associated with signal transduction were linked to later onset of seizures (e.g., “postsynaptic specialization,” 12.5 months; “dendritic spine,” 16 months) (Figure 2A). Biological processes linked to earlier onset mainly involved synaptic transmission (“regulation of presynaptic membrane potential”, median age = 2 months; “synaptic vesicle cycle”, 6.5 months) and protein synthesis (“tRNA aminoacylation for protein translation”, 4 months). Processes associated with neurogenesis and higher neuronal functions were linked to later onset (“learning or memory,” 13 months; “regulation of neurogenesis,” 14 months; “gliogenesis,” 19 months, “adult behavior,” 36 months) (Figure 2B). The three enriched GO‐MF terms, associated with protein synthesis and channel activities, were all associated with relatively early seizure onset (Figure 2C).

**FIGURE 2:**
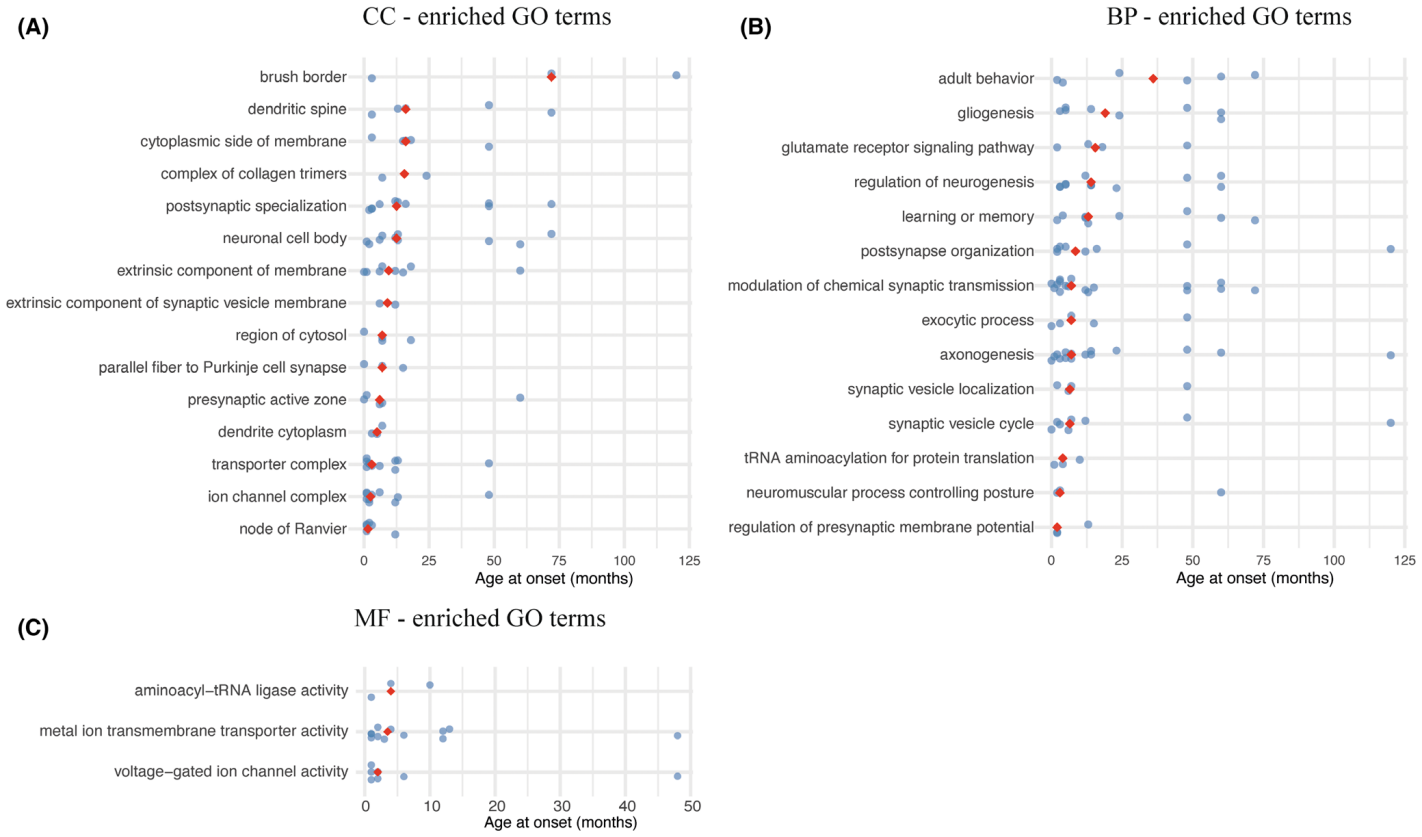
Enriched GO terms of the study cohort by age at onset of seizures. Dot plots illustrating significantly enriched GO terms across the study cohort, stratified by age at seizure onset. The y‐axis lists the enriched GO terms, while the x‐axis represents the age at seizure onset (in months) for genes associated with each term. Each red dot indicates the median seizure‐onset age of all patients carrying variants in genes contributing to that GO term. GO terms are ranked in descending order according to their median onset age. Enrichment results are displayed separately for the subontologies cellular component (CC) in panel (A), biological process (BP) in panel (B) and molecular function (MF) in panel (C).

The next objective was to evaluate associations between GO terms and the three clinically defined age groups. According to the previous analysis, genes with seizure onset at 0–3 months (group 1) were mainly enriched in GO‐CC terms attributable to axonal (“node of Ranvier”) and channel complex (“voltage‐gated sodium channel complex”). For genes linked to onset at 4–12 months (group 2), a spectrum of GO‐CC enrichments was observed predominantly involving synaptic compartments (e.g., “extrinsic component of synaptic vesicle membrane,” “dendrite cytoplasm”), but also including broader terms such as “region of cytosol” and “extrinsic component of membrane.” Genes with seizure onset at >12 months (group 3) exhibited a more heterogeneous distribution across cellular compartments, including synaptic compartments (e.g., “postsynaptic membrane,” “postsynaptic specialization”) but also compartments reflecting neuronal plasticity such as “dendritic spine” and “neuron spine” (Figure 3A).

**FIGURE 3:**
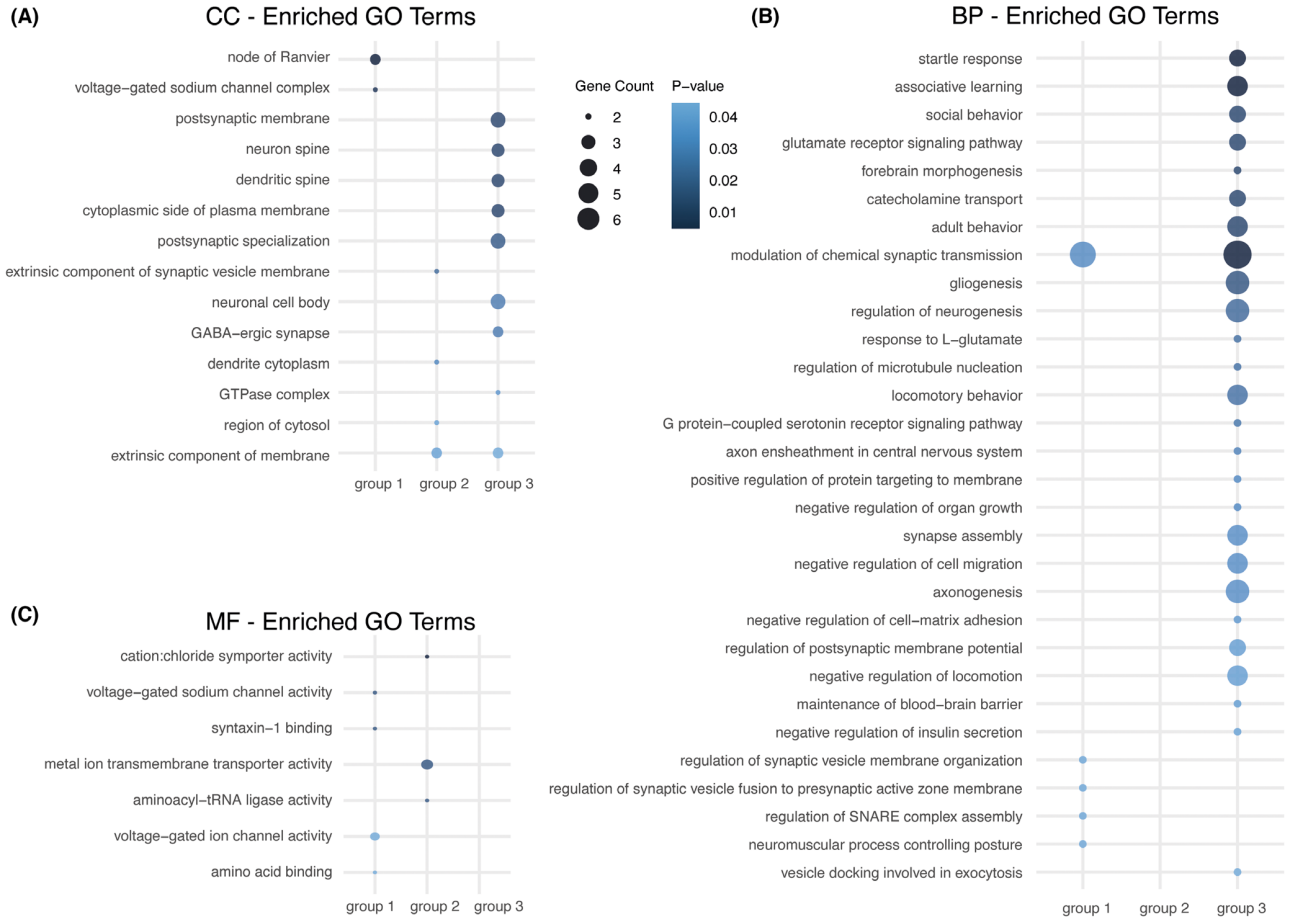
Enriched GO terms across different age groups. Enrichment plots illustrate significantly enriched GO terms identified among genes grouped by age at seizure onset. The y‐axis lists the enriched GO terms, while the x‐axis represents the distinct age groups. The size of each circle corresponds to the number of genes associated with a given GO term, and the color scale indicates the adjusted *p*‐value (fdr‐corrected significance level). Results are shown separately for the subontologies cellular component (CC) in panel (A), biological process (BP) in panel (B), and molecular function (MF) in panel (C).

For biological processes (GO‐BP), genes in group 1 were mainly enriched in synaptic transmission (“modulation of chemical synaptic transmission”) including especially presynaptic signaling processes (“regulation of SNARE complex assembly”, “regulation of synaptic vesicle membrane organization”, “regulation of synaptic vesicle fusion to presynaptic active zone membrane”). Group 2 did not demonstrate any specific enrichment pattern regarding the subcategory GO‐BP. Group 3 genes showed broad distribution, including synaptic transmission (e.g., “glutamate receptor signaling pathway”, “modulation of chemical synaptic transmission,” “regulation of postsynaptic membrane potential,” “synapse assembly”), neurodevelopmental programs (e.g., “negative regulation of cell migration,” “forebrain morphogenesis,” “regulation of neurogenesis,” “gliogenesis,” “axogenesis,” “regulation of microtuble nucleation”), and behavioral processes (e.g., “associative learning,” “social behavior,” “adult behavior”) (Figure 3B). For GO‐MF, enriched terms associated with ion channel and transporter activity were found in groups 1 and 2 but not group 3 (Figure 3C).

No significant differences in GO terms were found related to neurodevelopmental delay or MRI abnormalities.

## 4 DISCUSSION

We investigated genes associated with monogenic childhood epilepsy and identified common molecular mechanisms using GO enrichment analysis. A total of 66 different causative genes (class 4 or 5, ACMG) and four causative CNVs were identified by duo/trio whole‐exome sequencing in a cohort of 80 unrelated children with epilepsy according to ILAE criteria.

Our findings highlight a remarkable genetic diversity underlying childhood epilepsy. The most frequently identified genes (*KCNQ2*, *CDKL5, CHD2, MYT1L, PAK1, SCN8A, SLC12A9, STXBP1, TRIO*) corresponded to other pediatric epilepsy studies.^9^, ^25^, ^26^, ^27^, ^28^, ^29^ From a clinical perspective, we divided seizure onset into three categories: before 4 months of age (neonatal epilepsy); 4–12 months of age (typical IESS age), and toddler age and beyond. In our cohort, five genes (*CDKL5, STXBP1, SCN8A, TRIO, SLC6A1*) were identified across different seizure‐onset groups, indicating that identical genetic etiologies may manifest with variable phenotypic timing. Notably, the *SLC6A1* cases differed by only one month (12 vs. 13 months), reflecting separation by the predefined age threshold. Since we did not classify seizure and epilepsy types in this study, it remains unclear whether the epileptological manifestation was identical. Phenotypic heterogeneity has been described for genes including *SCN8A*, *STXBP1*, and *TRIO* with onset from neonatal to later childhood.^30^, ^31^, ^32^ Genes that cause characteristic clinical syndromes, such as *TSC1/2* (tuberous sclerosis), *UBE3A* (Angelman syndrome), *SCN1A* (Dravet syndrome), and *MECP2* (Rett syndrome), are significantly underrepresented in our cohort, as these are often diagnosed on the basis of clinical suspicion using single‐gene and gene panel testing rather than WES.

The high proportion of de novo autosomal dominant variants mirrors findings from other sequencing studies of early‐onset epileptic encephalopathies and reinforces the importance of trio‐based approaches in diagnostics, as the index‐parent comparison improves the detection and interpretation of de novo variants.^26^, ^33^, ^34^, ^35^ Most pathogenic variants are missense variants, which is consistent with previous studies and underscores the importance of understanding the functional impact of missense variants. Depending on the affected gene and variant, these may include gain‐of‐function, loss‐of‐function, dominant‐negative, or other variant‐specific effects, which are relevant for mechanistic understanding and might be crucial for treatment decisions.^2^ From a clinical perspective, most children with epilepsy in our cohort exhibited neurodevelopmental delay, and nearly half showed structural abnormalities on MRI, which is likely biased due to our selection process for WES.

GO enrichment analysis identified overrepresented cellular components, biological processes, and molecular functions compared to all brain‐expressed genes. Biological processes were enriched for synaptic function regulation, neurodevelopmental programs, including neurogenesis and neuronal differentiation, and higher‐order neuronal activity. Cellular components corresponded to synaptic compartments and channel‐ and transporter‐associated membrane structures. At the molecular function level, we further identified significant enrichment of ion channel–related terms as well as protein biosynthesis–associated functions, linking membrane excitability and translational regulation to the underlying synaptic and developmental mechanisms.

These findings align with large‐scale genomic studies in epilepsy. The Epi25 study highlighted “synaptic transmission & membrane excitability”^2^ and the ILAE Consortium reported enrichment of genes regulating synaptic processes in excitatory and inhibitory neurons.^36^ Notably, our observation of enrichment for neurogenesis and neuronal differentiation is corroborated by single‐cell analyses in neurodevelopmental disorders, which identified convergent defects in neural progenitor cells and their cell‐type transitions.^37^ These findings demonstrate that epilepsy genes are enriched in synaptic signaling pathways but also in developmental processes including neuronal differentiation and cell cycle regulation, indicating that early neurodevelopmental disruptions may predispose to network dysfunction. Age‐specific GO term enrichment revealed that genes associated with very early onset of epilepsy are enriched for early neurodevelopmental programs, including synaptic transmission and protein biosynthesis. These processes establish neuronal architecture and network connectivity during pre‐ and postnatal development.^25^, ^28^, ^34^, ^38^, ^39^, ^40^, ^41^, ^42^ Disruption impairs circuit formation and stability, predisposing to early‐life seizures. At a molecular function level (GO‐MF), early‐onset genes (group 1 and group 2) were enriched for ion channel‐related and protein biosynthesis‐associated functions, linking membrane excitability and translational regulation to the developmental processes identified at the biological process level. This is consistent with bioinformatic analyses demonstrating enrichment of synaptic signaling, neuronal excitability, and neurodevelopmental pathways, as well as ion channel activity and aminoacyl‐tRNA ligase functions.^25^, ^34^ Experimental data further support the critical role of voltage‐gated channels in early neuronal differentiation and network formation.^41^ Consistent with this, early‐onset cases show GO‐CC associations with axonal compartments and trafficking processes, while synaptic signaling enrichment is prominent beyond the neonatal period (from group 2 onwards). This aligns with evidence that defects in presynaptic vesicle trafficking and release, including SNARE‐complex and SV2A‐related proteins, drive severe early epileptic encephalopathies.^25^, ^28^, ^38^, ^40^, ^43^ Multicenter clinical‐genetic studies further corroborate the enrichment of genes affecting synaptic vesicle cycling (such as *STXBP1*), ion channel complexes (including *SCN8A*), and early neurodevelopmental mechanisms (such as *CDKL5*).^39^ Genetic analyses of infantile epileptic spasms syndrome further support the involvement of neuronal migration, axonal and dendritic development, synaptogenesis, and later synaptic function.^43^, ^44^ In contrast, genes associated with slightly later onset of epilepsy show a shift toward biological processes involved in neuronal maturation, including neuronal plasticity and synaptic refinement, which become increasingly important during postnatal brain development and continue into adolescence and young adulthood. GO‐CC enrichment supported this shift, with overrepresentation of “postsynaptic specialization,” “dendritic spine,” and “neuron spine.” These structures are central to activity‐dependent synaptic refinement and structural remodeling, underscoring the increasing importance of neuronal plasticity during later stages of brain maturation. Interestingly, the GO term “brush border” is associated with a comparable early, but also the latest median onset of seizures in our cohort. Although *ACTB, SHANK2*, and *DNM1L*, which carry this GO term, have distinct primary functions—*ACTB* encoding β‐actin as a core cytoskeletal component, *DNM1L* mediating mitochondrial fission and energy homeostasis, and *SHANK2* acting as a scaffolding protein of the postsynaptic density—they functionally converge on the maintenance of neuronal morphology, synaptic organization, and energy‐dependent plasticity.^45^, ^46^, ^47^ Disruption of these mechanisms impairs neuronal connectivity and synaptic stability, contributing to epileptogenesis.^48^

Overall, stratification of patients by age at seizure onset reveals biologically meaningful differences indicating a shift from disturbances of early brain construction toward impairments of mature neuronal function as key determinants of seizure onset timing.

Compared to other studies using GO enrichment, it should be noted that our analyses related enrichments only to genes expressed in the brain, implicating more specific results.

There are limitations to our study. Despite substantial cohort size for a single‐center study, genetic heterogeneity limited statistical power, as most genes appeared in single individuals. Due to the small sample size, GO terms from higher hierarchy levels had to be used; for large groups, the categories (CC, MF, BP) could be further subdivided. In addition, the age grouping was based on clinical judgment and may introduce a certain ascertainment bias. It should also be noted that we did not classify different sub‐cohorts according to seizure type, as seizure semiology often changes during childhood and is subject to developmental dynamics. Furthermore, the cohort is not representative of pediatric epilepsies in general, as inclusion in the sequencing cohort was driven by the presence of a severe, presumably genetic neurodevelopmental disorder requiring duo−/trio‐ whole exome analysis. The epilepsy cohort analyzed here therefore represents a selected subgroup, and common self‐limiting or relatively benign epilepsy syndromes were not included.

In conclusion, our findings highlight the potential of GO enrichment analysis to identify common mechanisms associated with epilepsy even in very heterogeneous groups.

The integration of phenotypic, genotypic, and functional enrichment data in larger sample sizes might provide insights into the molecular principles of childhood epilepsy and help to identify common as well as distinct mechanisms depending on the kind of epilepsy, age of onset, and comorbidity, with potential relevance for future therapeutic strategies.

## AUTHOR CONTRIBUTIONS

Laura Hecher: Conceptualization, Methodology, Investigation, Data curation, Validation, Visualization, Writing – original draft, Writing – review & editing. Mathias Woidy: Methodology, Formal analysis, Data curation, Visualization, Writing – original draft, Writing – review & editing. Franziska Plein: Investigation, Data curation, Writing – review & editing. Tatjana Bierhals: Investigation, Writing – review & editing. Leonie von Elsner: Investigation, Writing – review & editing. Theresia Herget: Investigation, Writing – review & editing. Maja Hempel: Investigation, Data curation, Writing – review & editing. Fanny Kortum: Investigation, Data curation, Validation, Writing – review & editing. Lev Grinstein: Investigation, Writing – review & editing. Julia V. Cramer: Investigation, Writing – review & editing. Jessika Johannsen: Investigation, Writing – review & editing. Jonas Denecke: Conceptualization, Methodology, Investigation, Writing – original draft, Writing – review & editing, Supervision.

## CONFLICT OF INTEREST STATEMENT

None of the authors has any conflict of interest to disclose. We confirm that we have read the Journal’s position on issues involved in ethical publication and affirm that this report is consistent with those guidelines.

## Supporting information


**Table S1:** Detailed clinical and genetic characteristics of the cohort of 80 patients with epilepsy. The table provides data on sex, age at examination, age at seizure onset, neurodevelopmental delay, and abnormalities on brain magnetic resonance imaging (MRI). Genetic characteristics include the associated gene, its classification at the time of diagnosis as an established OMIM disease gene, candidate gene, or newly identified disease gene, mode of inheritance, and variant type.

**Table S2:** Significantly enriched Gene Ontology terms and associated genes. Gene Ontology (GO) enrichment analysis of the disease‐associated genes in the study cohort. The table includes all significantly enriched GO terms across the three GO domains: biological process (BP), cellular component (CC), and molecular function (MF). For each GO term, the GO identifier (GO ID), p‐value, gene ratio, fold enrichment (FE), gene count, and associated genes are provided.

## Data Availability

The data that support the findings of this study are available from the corresponding author upon reasonable request. The data are not publicly available due to privacy and ethical restrictions.
